# Supplementary material for: A Mathematical Model to Estimate Chemotherapy Concentration at the Tumor-Site and Predict Therapy Response in Colorectal Cancer Patients with Liver Metastases
Source: Cancers (Basel). 2021 Jan 25;13(3):444. doi: 10.3390/cancers13030444 (PMC7866038; doi:10.3390/cancers13030444)
Supplement: Supplementary file 1 [file cancers-13-00444-s001.pdf]

SUPPLEMENTAL INFORMATION

A mathematical model to estimate chemotherapy concentration at the tumor-site and predict therapy response in colorectal cancer patients with liver metastases

Daniel A. Anaya, Prashant Dogra, Zhihui Wang, Mintallah Haider, Jasmina Ehab, Daniel K. Jeong, Masoumeh Ghayouri, Gregory Y. Lauwers, Kerry Thomas, Richard Kim, Joseph D. Butner, Sara Nizzero, Javier Ruiz Ramírez, Marija Plodinec, Richard L. Sidman, Webster K. Cavenee, Renata Pasqualini, Wadih Arap, Jason B. Fleming, Vittorio Cristini

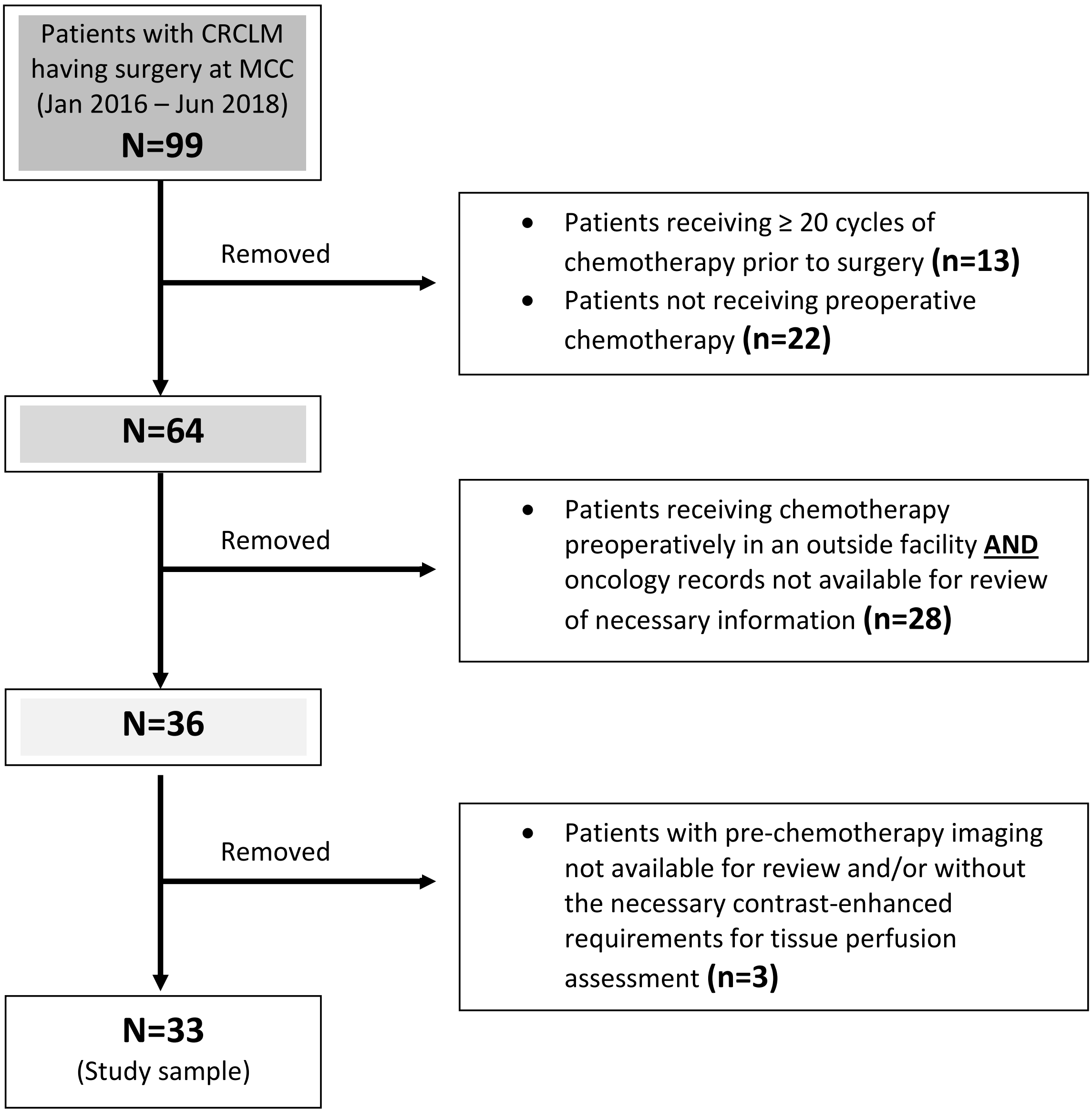

Supplemental Figure S1. Workflow describing patient selection criteria (N=33).

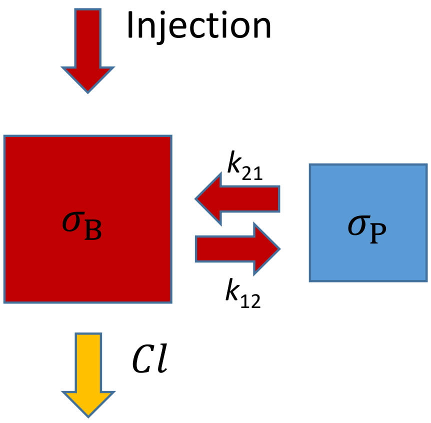

Supplemental Figure S2. Two-compartment pharmacokinetic model.

The model consists of a central and a peripheral compartment characterized by σ_{B} and σ_{P}, respectively, which are the concentrations of the injected drug in central and peripheral compartment, respectively. k_{12} and k_{21} represent the first order intercompartment mass transfer rate constants, and Cl is the clearance of the drug from the central compartment.

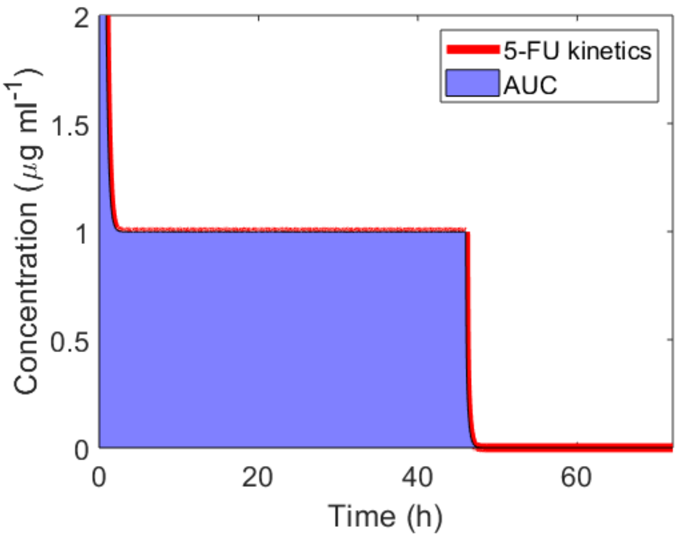

Supplemental Figure S3. Systemic concentration kinetics of 5-FU.

Numerical solution of the 2-compartment PK model for a single therapy cycle containing concomitant administration of an intravenous bolus and intravenous infusion of 5-FU. The red curve represents the concentration kinetics of 5-FU and the light blue area represents the area under the concentration-time curve (AUC).

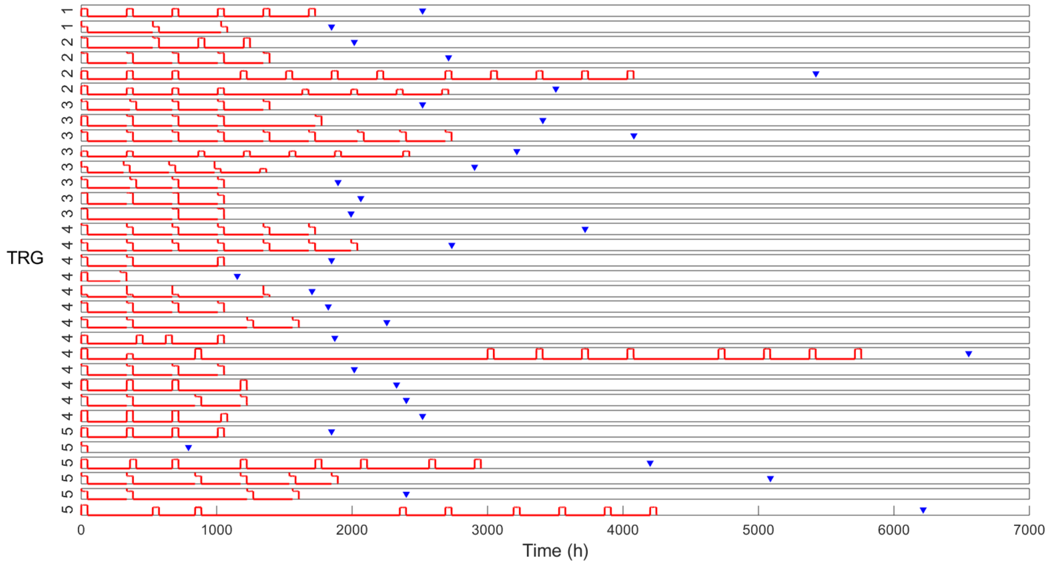

Supplemental Figure S4. Complete chemotherapy regimen.

Systemic concentration kinetics of 5-FU over multiple therapy cycles is shown. A master plot containing subplots (n = 33) where each subplot shows the concentration-time kinetics of 5-FU for an individual patient, over the duration of treatment. The y-axis of each subplot ranges from 0-2 µg·ml^{-1}. Subplots are sorted in an increasing order of the corresponding TRG values. Inverted blue triangles indicate the time of TRG assessment (or surgery).

Supplemental Table S1. Parameter estimates of multivariable logistic (ordinal) regression

	**TRG = 1 vs TRG >1**	**TRG ≤ 2 vs TRG >2**	**TRG ≤ 3 vs TRG >3**	**TRG ≤ 4 vs TRG >4**
$\boldsymbol{\alpha_i}$	-3.49 (*P* > 0.05)	-2.2 (*P* > 0.05)	-0.97 (*P* > 0.05)	0.94 (*P* > 0.05)
$\boldsymbol{\beta_1}$ **(age)**	0.02 (*P* > 0.05)	0.02 (*P* > 0.05)	0.02 (*P* > 0.05)	0.02 (*P* > 0.05)
$\boldsymbol{\beta_2}$ **(gender)**	-0.03 (*P* > 0.05)	-0.03 (*P* > 0.05)	-0.03 (*P* > 0.05)	-0.03 (*P* > 0.05)
$\boldsymbol{\beta_3}$ **(presentation)**	-0.3 (*P* > 0.05)	-0.3 (*P* > 0.05)	-0.3 (*P* > 0.05)	-0.3 (*P* > 0.05)
$\boldsymbol{\beta_4}$ **(# lesions)**	-0.1 (*P* > 0.05)	-0.1 (*P* > 0.05)	-0.1 (*P* > 0.05)	-0.1 (*P* > 0.05)
$\boldsymbol{\beta_5}$ **(extrahepatic disease)**	-0.3 (*P* > 0.05)	-0.3 (*P* > 0.05)	-0.3 (*P* > 0.05)	-0.3 (*P* > 0.05)
$\boldsymbol{\beta_6}$ **(location)**	0.3 (*P* > 0.05)	0.3 (*P* > 0.05)	0.3 (*P* > 0.05)	0.3 (*P* > 0.05)

Corresponding *P* values of the estimates are given in parenthesis
